# Trends in adult current asthma prevalence and contributing risk factors in the United States by state: 2000–2009

**DOI:** 10.1186/1471-2458-13-1156

**Published:** 2013-12-10

**Authors:** Xingyou Zhang, Teresa Morrison-Carpenter, James B Holt, David B Callahan

**Affiliations:** 1 Division of Population Health, National Center for Chronic Disease Prevention and Health Promotion, Centers for Disease Control and Prevention, 4770 Buford Highway, Mailstop F78, Atlanta 30341, GA USA; 2 Centers for Disease Control and Prevention, National Center for Environmental Health, Air Pollution and Respiratory Health Branch, Atlanta GA, USA

**Keywords:** Current asthma, Smoking, Obesity, Trend, The United States

## Abstract

**Background:**

Current asthma prevalence among adults in the United States has reached historically high levels. Although national-level estimates indicate that asthma prevalence among adults increased by 33% from 2000 to 2009, state-specific temporal trends of current asthma prevalence and their contributing risk factors have not been explored.

**Methods:**

We used 2000–2009 Behavioral Risk Factor Surveillance System data from all 50 states and the District of Columbia (D.C.) to estimate state-specific current asthma prevalence by 2-year periods (2000–2001, 2002–2003, 2004–2005, 2006–2007, 2008–2009). We fitted a series of four logistic-regression models for each state to evaluate whether there was a statistically significant linear change in the current asthma prevalence over time, accounting for sociodemographic factors, smoking status, and weight status (using body mass index as the indicator).

**Results:**

During 2000–2009, current asthma prevalence increased in all 50 states and D.C., with significant increases in 46/50 (92%) states and D.C. After accounting for weight status in the model series with sociodemographic factors, and smoking status, 10 states (AR, AZ, IA, IL, KS, ME, MT, UT, WV, and WY) that had previously shown a significant increase did not show a significant increase in current asthma prevalence.

**Conclusions:**

There was a significant increasing trend in state-specific current asthma prevalence among adults from 2000 to 2009 in most states in the United States. Obesity prevalence appears to contribute to increased current asthma prevalence in some states.

## Background

Current asthma prevalence among adults in the United States has reached historically high levels. In 2009, 8.4% of U.S. adults (19.5 million persons) reported asthma compared with 7.2% (14.7 million persons) in 2000 [1,2]. Although national-level estimates indicate that asthma prevalence among adults relatively increased by 33% (4.8 million persons) from 2000 to 2009 (almost 500,000 adults per year) [1,2], state specific temporal tends in asthma prevalence and their contributing risk factors have not been explored.

To determine the prevalence of current asthma in the United States each year, the Centers for Disease Control and Prevention (CDC) uses national and state-based surveillance systems. Since the late 1970s, the National Health Interview Survey (NHIS), a national-based survey of households from all 50 states and the District of Columbia (D.C.), has provided a national estimate of asthma prevalence by population-based characteristics each year in the United States [3]. However, a systematic state-based survey, the Behavioral Risk Factor Surveillance System (BRFSS), provides state-level estimates of asthma prevalence by population-based characteristics each year.

Estimates of current asthma prevalence from both the national- (NHIS) and the state-based (BRFSS) surveys show that current asthma prevalence among adults is increasing [1,2,4-6] and continuing to vary by certain sociodemographic, behavioral, and geographic factors [7-12]. More specifically, asthma prevalence can vary by certain modifiable (e.g., weight and smoking status) [13-25] and non-modifiable (e.g., age and race status) risk factors [7-11,26-30]; however, the extent to which these risk factors contribute to increasing prevalence at the state level over time has not been analyzed or determined. Identifying state-specific changes in asthma prevalence and determining how known risk factors for asthma contribute to changes in prevalence over time are important for public health planning and for generating hypotheses for asthma prevention and control.

## Methods

### Sample population

We used cross-sectional data from the 2000–2009 BRFSS to estimate state-specific prevalence in current asthma and evaluate the influence of risk factors on current asthma prevalence [31]. BRFSS is a state-based random-digit dialing telephone survey that annually collects information on health conditions and risk behaviors of adults 18 years and older noninstitutionalized U.S. population in all 50 states, D.C., and U.S. territories. It uses a disproportionate stratified sample (DSS) design that commonly divides telephone numbers into two strata for sampling and also disproportionately samples smaller geographically defined populations of interest within a state. Further details on BRFSS survey methods and data are available at http://www.cdc.gov/brfss/index.htm.

### Measures

The outcome measure is current asthma status (yes or no). In the 2000 BRFSS, all respondents were asked, “Did a doctor ever tell you that you had asthma?” From 2001 to 2009, the BRFSS question was changed and all respondents were asked, “Have you ever been told by a doctor, nurse, or other health professional that you had asthma?” From 2000 to 2009, if the respondent confirmed that they had been told they had asthma, they then were asked, “Do you still have asthma?” We defined current asthma as an affirmative response to both questions. To reduce the annual variability in survey measures [32], we combined two consecutive survey years and categorized “year of survey” into five 2-year periods: 2000–2001, 2002–2003, 2004–2005, 2006–2007, and 2008–2009.

To evaluate the effect of known risk factors on current asthma prevalence, we included BRFSS self-reported sociodemographic factors (age, sex, race/ethnicity, education, and household income) and modifiable behaviors (smoking status and weight status) associated with current asthma prevalence and available from BRFSS [31]. Age was categorized into six groups: 18–24, 25–34, 35–44, 45–54, 55–64, and ≥65 years. Race and ethnicity were categorized as non-Hispanic white, non-Hispanic black, Hispanic, and other. Education was categorized as less than high school, completion of high school, some college (including associate’s degree), and a bachelor's degree or higher. Household income was categorized as: <$15,000, $15,000–$24,999, $25,000–$49,999, $50,000–$74,999, ≥$75,000, or missing. Missing income values were included as a separate category because 13% of records in the 2000–2009 BRFSS did not specify income values. We categorized smoking status into three groups: current smokers (respondents who had smoked at least 100 cigarettes in their entire life and currently smoke), previous smokers (respondents who had smoked at least 100 cigarettes in their entire life but no longer smoke), and nonsmokers (respondents who had never smoked or had smoked fewer than 100 cigarettes in their life). To classify a respondent’s weight status, we used body mass index (BMI; calculated as respondent’s reported weight in kilograms divided by respondent’s reported height in meters squared). Using BMI, we categorized weight status into five groups: underweight (BMI <18.5 kg/m^2^), healthy weight (18.5 kg/m^2^ ≤ BMI < 25.0 kg/m^2^), overweight (25.0 kg/m^2^ ≤ BMI < 30.0 kg/m^2^), obese (30.0 kg/m^2^ ≤ BMI < 40.0 kg/m^2^), and morbidly obese (BMI ≥40.0 kg/m^2^) [33].

### Statistical analysis

All statistical data analyses were conducted using SAS-Callable SUDAAN software (Version 10, Research Triangle Institute, NC). The BRFSS complex survey design structures were considered in all current asthma outcome estimates by state, year of survey, sociodemographic factors (sex, age, race/ethnicity, household income, and education), smoking status, and weight status; BRFSS final post-stratified sampling weights were used in all statistical data analyses. We reported weighted percentages with 95% confidence intervals (CIs) for all population or subpopulation groups for current asthma prevalence estimates. For all analyses, we considered a P value of <0.05 significant. We fitted a series of four logistic regression models to examine temporal changes in asthma prevalence from 2000 to 2009 for the United States, each state, and D.C. The first model (model I) included only the continuous variable of 2-year survey periods to evaluate the linear trend in the prevalence of current asthma over the time period from 2000–2001 to 2008–2009. The second model (model II) adjusted for sociodemographic factors (age, sex, race/ethnicity, education, and household income) to evaluate whether an increasing linear trend in current asthma prevalence still existed. The third model (model III) introduced smoking status to evaluate its impact on the linear trends in current asthma prevalence after adjusting for individual sociodemographic factors. The fourth model (model IV) introduced weight status and evaluated its effect on the linear trends in current asthma prevalence while controlling for individual sociodemographic factors and smoking status. We used the model-based predicted marginal prevalence ratios (PRs) and prevalence differences (PDs) and P values from their related t-Test to assess the direction and significance of linear trends in current asthma prevalence after controlling for the temporal changes in population sociodemographics, smoking, and weight status within a state. The model-adjusted prevalence differences for survey periods 2000–2001 compared to 2008–2009 were used to evaluate the increasing magnitude of asthma prevalence [34].

## Results

From 2000 to 2009, BRFSS collected records on 3,203,280 respondents from the 50 states and D.C. Of these respondents, 159,407 (5.0%) were excluded from the analysis because values for asthma status or sociodemographic factors other than household income, smoking status, or weight status were missing; 3,043,873 respondents were included in the analysis. The average sample size per year from 2000 to 2009 was 304,387 (range: from 174,810 in 2000 to 411,406 in 2007) and per state was 6,000 (range: from 4,621 in WY to 16,112 in WA). Survey response rates varied by year and state; the median state survey response rate for this time period was a little more than 50% (detailed at http://www.cdc.gov/brfss/technical_infodata/quality.htm).

### Current asthma prevalence

Table 1 provides a summary of current asthma prevalence and unadjusted and adjusted prevalence ratios by 2-year periods, sociodemographic factors, smoking status, and weight status. Current asthma prevalence relatively increased by 18.2% nationally from 7.2% during 2000–2001 to 8.5% during 2008–2009. Population subgroups with asthma prevalence greater than the 2008–2009 national level of 8.5% include persons who are: female (9.8%), aged 18–24 years (9.4%), black, non-Hispanics (9.3%); persons with: less than high school education (9.3%), household income < $15,000 (11.6%), household income $15,000-$25,000 (9.1%); and persons who: currently smoke (9.2%), are obese (10.2%), or are morbidly obese (18.2%). Population subgroups with prevalence ratio (PR) estimates >1.5 for current asthma include persons: who are female (PR: 1.69, 95% CI: 1.66-1.72, compared to males); have household incomes < $15,000 (PR: 1.58, 95% CI: 1.53-1.64, compared to ≥ $75,000); are obese (PR: 1.63, 95% CI: 1.60-1.67, compared to healthy weight); or are morbidly obese (PR: 2.60, 95% CI: 2.52-2.68, compared to healthy weight).

**Table 1:** U.S. adult current asthma prevalence and prevalence ratios (PR) by 2000–2009 BRFSS sample characteristics

**Characteristics**	**Subpopulation**	**Sample size**	Prevalence*	**Unadjusted**	**Adjusted**^**#**^
	**Groups**	**n = 3,043,873**	**% (95% CI**^**†**^**)**	**PR (95% CI**^**†**^**)**	**PR (95% CI**^**†**^**)**
2-Year	2000–2001	374,137	7.21 (7.07, 7.35)	Reference	
Period	2002–2003	486,368	7.60 (7.47, 7.74)	1.04 (1.04, 1.05)	1.04 (1.04, 1.05)
	2004–2005	629,633	7.99 (7.86, 8.12)	1.09 (1.07, 1.10)	1.09 (1.07, 1.10)
	2006–2007	748,536	8.23 (8.10, 8.37)	1.13 (1.11, 1.15)	1.13 (1.11, 1.15)
	2008–2009	805,199	8.52 (8.40, 8.65)	1.18 (1.15, 1.20)	1.18 (1.15, 1.20)
Sex	Male	1,176,599	5.90 (5.82, 5.99)	Reference	
	Female	1,867,274	9.83 (9.75, 9.92)	1.67 (1.64, 1.69)	1.69 (1.66, 1.72)
Age,	18 – 24	159,715	9.36 (9.11, 9.62)	Reference	
in years	25 – 34	388,151	7.65 (7.51, 7.80)	0.82 (0.79, 0.84)	0.79 (0.76, 0.82)
	35 – 44	537,390	7.44 (7.32, 7.56)	0.79 (0.77, 0.82)	0.74 (0.72, 0.76)
	45 – 54	623,754	7.98 (7.85, 8.10)	0.85 (0.83, 0.88)	0.75 (0.73, 0.78)
	55 – 64	560,588	8.43 (8.29, 8.57)	0.90 (0.87, 0.93)	0.75 (0.73, 0.77)
	≥ 65	774,275	7.28 (7.17, 7.40)	0.78 (0.75, 0.80)	0.63 (0.61, 0.65)
Race and	White (NH)**	2,451,608	8.09 (8.03, 8.16)	Reference	
Ethnicity	Black (NH)	234,974	9.26 (9.04, 9.49)	1.15 (1.12, 1.17)	0.96 (0.94, 0.99)
	Hispanic	188,189	5.86 (5.66, 6.06)	0.72 (0.70, 0.75)	0.64 (0.62, 0.67)
	Others	169,102	8.37 (8.09, 8.66)	1.03 (1.00, 1.07)	1.05 (1.01, 1.08)
Education	< High school	310,985	9.26 (9.06, 9.46)	1.32 (1.28, 1.35)	1.12 (1.09, 1.16)
	High school	936,827	7.85 (7.74, 7.96)	1.12 (1.10, 1.14)	0.93 (0.91, 0.95)
	Some college	815,293	8.48 (8.36, 8.60)	1.21 (1.18, 1.23)	1.02 (1.00, 1.04)
	>Bachelor degree	980,768	7.03 (6.93, 7.12)	Reference	
Household	< $15, 000	301,442	11.6 (11.4, 11.9)	1.74 (1.69, 1.79)	1.58 (1.53, 1.64)
Income	$15 k^##^ ~ <$25 k	474,354	9.06 (8.90, 9.23)	1.35 (1.32, 1.39)	1.27 (1.24, 1.31)
	$25 k ~ <$50 k	811,140	7.54 (7.42, 7.65)	1.13 (1.10, 1.15)	1.07 (1.05, 1.10)
	$50 k ~ <$75 k	449,348	7.05 (6.91, 7.20)	1.05 (1.03, 1.08)	1.01 (0.98, 1.04)
	≥$75 k	611,531	6.69 (6.57, 6.81)	Reference	
	Unknown	396,058	7.99 (7.81, 8.16)	1.19 (1.16, 1.23)	1.15 (1.11, 1.18)
Smoking	Never	1,595,769	7.23 (7.15, 7.31)	Reference	
Status	Former	853,780	8.41 (8.29, 8.52)	1.16 (1.14, 1.18)	1.24 (1.22, 1.26)
	Current	594,324	9.21 (9.07, 9.35)	1.27 (1.25, 1.3)	1.26 (1.24, 1.29)
Weight	Underweight	50,089	8.70 (8.22, 9.20)	1.29 (1.22, 1.37)	1.10 (1.04, 1.17)
Status	Healthy weight	1,063,457	6.75 (6.66, 6.85)	Reference	
	Overweight	1,064,475	6.91 (6.81, 7.00)	1.02 (1.00, 1.04)	1.18 (1.15, 1.20)
	Obese	639,896	10.2 (10.0, 10.3)	1.51 (1.48, 1.54)	1.63 (1.60, 1.67)
	Morbidly obese	96,785	18.2 (17.7, 18.7)	2.69 (2.62, 2.78)	2.60 (2.52, 2.68)

*Weighted prevalence for complex survey design; ^#^Adjusted by logistic regression for all factors listed; ^†^CI = Confidence Interval;

**NH = Non-Hispanic; ^##^k = thousand.

### Trends in current asthma prevalence

**Figure 1:**
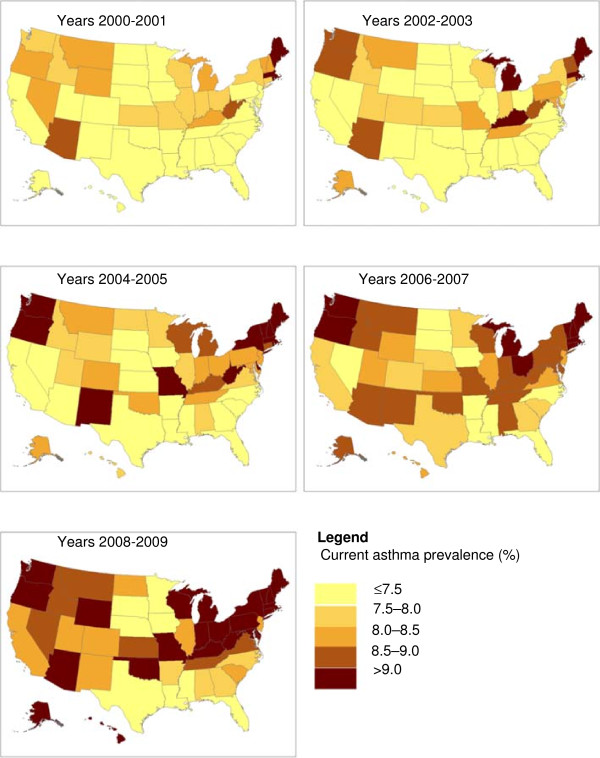
Current asthma unadjusted prevalence by state.

All 50 states and D.C. showed an increase in current asthma unadjusted prevalence from 2000–2001 to 2008–2009; and over the 10-year period, current asthma prevalence increased by >2.0 percentage points in 9 states (AK, DC, HI, LA, NH, NJ, OK, PA, and SD) and by 1.5-2.0 percentage points in 12 states (DE, KY, MD, MI, NM, NY, OH, OR, TN, VA, VT, and WI) (Figure 1 and Table 2).

**Table 2:** Average 2-year current asthma unadjusted prevalence among adults by state, BRFSS, 2000–2009

**State**	**2-year period**				
	**2000–2001**	**2002–2003**	**2004–2005**	**2006–2007**	**2008–2009**
	% (se*)	% (se*)	% (se*)	% (se*)	% (se*)
US	7.21 (0.07)	7.60 (0.07)	7.99 (0.07)	8.23 (0.07)	8.52 (0.07)
AK	7.17 (0.58)	8.30 (0.60)	8.48 (0.58)	8.64 (0.57)	9.23 (0.67)
AL	6.23 (0.38)	7.33 (0.38)	7.91 (0.40)	8.74 (0.44)	7.71 (0.37)
AR	6.81 (0.39)	7.46 (0.35)	7.45 (0.33)	7.24 (0.31)	7.93 (0.43)
AZ	8.66 (0.71)	8.65 (0.54)	7.36 (0.47)	8.85 (0.53)	10.2 (0.67)
CA	7.23 (0.32)	7.44 (0.33)	7.46 (0.32)	7.64 (0.31)	8.20 (0.23)
CO	7.36 (0.45)	7.95 (0.35)	8.48 (0.33)	7.86 (0.27)	8.18 (0.26)
CT	7.91 (0.31)	8.44 (0.32)	8.97 (0.35)	9.27 (0.34)	9.14 (0.43)
DC	7.62 (0.52)	8.60 (0.56)	9.10 (0.50)	9.65 (0.45)	10.0 (0.51)
DE	7.37 (0.46)	7.49 (0.40)	9.11 (0.47)	8.71 (0.46)	9.19 (0.55)
FL	5.71 (0.27)	6.29 (0.32)	7.03 (0.29)	6.71 (0.23)	6.97 (0.33)
GA	6.76 (0.36)	7.23 (0.34)	7.35 (0.38)	7.84 (0.30)	7.82 (0.45)
HI	7.36 (0.38)	6.27 (0.31)	7.53 (0.45)	8.02 (0.35)	9.49 (0.38)
IA	6.58 (0.34)	6.36 (0.34)	6.95 (0.31)	6.74 (0.31)	7.20 (0.32)
ID	7.83 (0.32)	7.83 (0.33)	7.64 (0.32)	8.91 (0.39)	8.62 (0.39)
IL	7.89 (0.41)	7.29 (0.28)	7.74 (0.34)	8.32 (0.36)	8.47 (0.36)
IN	7.85 (0.37)	7.78 (0.28)	8.28 (0.29)	8.52 (0.33)	9.18 (0.38)
KS	7.98 (0.34)	7.60 (0.32)	7.18 (0.24)	8.34 (0.30)	8.64 (0.27)
KY	8.01 (0.31)	9.67 (0.38)	8.53 (0.37)	8.55 (0.37)	9.87 (0.39)
LA	5.07 (0.25)	6.10 (0.28)	6.08 (0.31)	6.12 (0.29)	7.22 (0.32)
MA	9.03 (0.28)	9.44 (0.31)	9.68 (0.32)	9.89 (0.27)	10.3 (0.29)
MD	7.27 (0.39)	8.13 (0.39)	8.13 (0.33)	8.66 (0.32)	9.25 (0.34)
ME	9.12 (0.49)	9.99 (0.50)	9.85 (0.44)	9.96 (0.38)	10.6 (0.35)
MI	8.19 (0.42)	9.04 (0.37)	8.67 (0.29)	9.50 (0.35)	9.98 (0.32)
MN	6.89 (0.35)	7.15 (0.34)	7.98 (0.41)	7.72 (0.38)	7.22 (0.37)
MO	7.78 (0.40)	8.27 (0.41)	9.05 (0.42)	8.62 (0.43)	9.01 (0.46)
MS	6.14 (0.41)	6.54 (0.32)	7.09 (0.32)	6.74 (0.29)	7.34 (0.28)
MT	8.07 (0.47)	8.41 (0.43)	8.30 (0.37)	8.72 (0.37)	8.89 (0.36)
NC	6.69 (0.37)	6.78 (0.32)	7.02 (0.20)	7.31 (0.23)	7.71 (0.26)
ND	7.20 (0.44)	7.18 (0.38)	7.54 (0.39)	7.33 (0.39)	8.42 (0.41)
NE	6.22 (0.37)	7.16 (0.32)	6.83 (0.28)	7.76 (0.38)	7.39 (0.33)
NH	8.42 (0.44)	8.59 (0.33)	10.3 (0.36)	10.0 (0.36)	10.4 (0.39)
NJ	6.19 (0.29)	7.44 (0.41)	8.09 (0.22)	8.14 (0.30)	8.23 (0.28)
NM	6.95 (0.35)	7.36 (0.32)	9.12 (0.34)	8.52 (0.35)	8.49 (0.35)
NV	8.31 (0.56)	7.16 (0.47)	7.08 (0.50)	7.39 (0.45)	8.71 (0.55)
NY	7.62 (0.37)	7.81 (0.31)	9.18 (0.32)	8.71 (0.33)	9.36 (0.35)
OH	7.96 (0.43)	7.20 (0.35)	8.17 (0.41)	9.37 (0.48)	9.83 (0.33)
OK	6.67 (0.31)	7.34 (0.26)	8.41 (0.28)	8.80 (0.30)	9.48 (0.33)
OR	8.21 (0.38)	8.94 (0.38)	9.96 (0.31)	9.85 (0.40)	9.90 (0.45)
PA	7.01 (0.34)	8.10 (0.32)	8.45 (0.30)	8.97 (0.35)	9.20 (0.32)
RI	9.01 (0.39)	9.18 (0.38)	10.2 (0.43)	10.2 (0.45)	10.4 (0.42)
SC	6.63 (0.36)	5.94 (0.31)	7.07 (0.26)	7.61 (0.28)	8.02 (0.34)
SD	5.43 (0.26)	6.63 (0.32)	7.04 (0.30)	7.39 (0.34)	7.47 (0.35)
TN	7.06 (0.38)	8.11 (0.42)	8.26 (0.40)	8.57 (0.43)	8.57 (0.43)
TX	6.29 (0.26)	7.00 (0.28)	7.02 (0.28)	7.70 (0.38)	6.95 (0.29)
UT	7.42 (0.44)	7.64 (0.42)	7.96 (0.35)	8.35 (0.38)	8.19 (0.35)
VA	6.83 (0.44)	7.40 (0.36)	7.94 (0.36)	8.21 (0.41)	8.51 (0.51)
VT	8.02 (0.35)	8.59 (0.37)	9.09 (0.32)	9.46 (0.34)	9.90 (0.34)
WA	7.93 (0.34)	8.98 (0.29)	9.18 (0.19)	9.12 (0.19)	9.10 (0.20)
WI	7.77 (0.41)	7.99 (0.36)	8.89 (0.37)	8.95 (0.43)	9.53 (0.48)
WV	8.89 (0.43)	8.61 (0.38)	9.66 (0.41)	8.79 (0.41)	9.24 (0.38)
WY	8.47 (0.43)	7.37 (0.35)	7.76 (0.33)	8.45 (0.34)	9.12 (0.34)
Median	7.37 (0.46)	7.60 (0.32)	8.13 (0.33)	8.55 (0.37)	8.89 (0.36)

^*^se = standard error.

**Figure 2:**
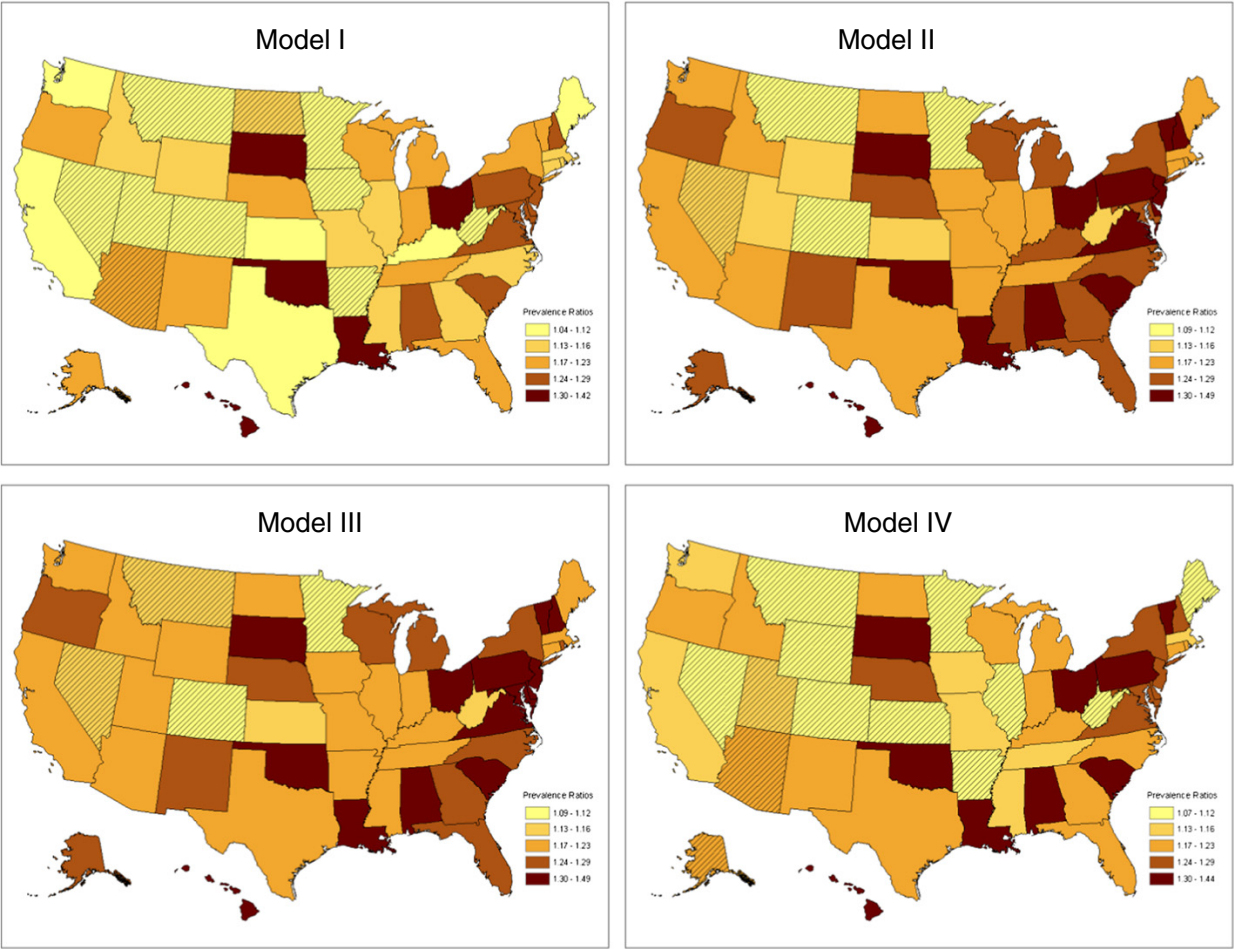
Current asthma prevalence ratios (PRs) for 2008–2009 vs 2000–2001.

In the unadjusted analysis comparing two time periods of current asthma prevalence, 2000–2001 to 2008–2009, 80% (40/50) of states and D.C. showed significant increases. Only ten states (AR, AZ, CO, IA, MN, MT, ND, NV, UT, and WV) showed increases in current asthma prevalence that were not statistically significant (Figure 2 and Table 3 [model I]). After controlling for survey year and changes in population sociodemographics, 46 states, including 6 of the 10 states (AR, AZ, IA, ND, UT, and WV) that showed insignificant increases in the unadjusted model (Figure 2 and [model I]), and D.C. showed a significant increase in current asthma prevalence in 2008–2009 compared to 2000–2001 (Figure 2 and Table 3 [model II]). When we added smoking status to the model with survey year and sociodemographic factors, MT shows a significant increase in current asthma prevalence and all other states keep unchanged in their linear trends of current asthma prevalence (Figure 2 and Table 3 [model III]) was observed except MT. However, when we added weight status to the model with survey year, sociodemographic factors, and smoking status, 10 states (AR, AZ, IA, IL, KS, ME, MTUT, WV , and WY,) that had previously shown a significant increasing trend showed no significant increasing linear trend in current asthma prevalence from 2000 to 2009 (Figure 2 and Table 3 [model IV]).

**Table 3:** Current asthma prevalence ratios (PR) for 2008–2009 vs 2000–2001 and significance by state

**State**	**Model I**	**Model II**	**Model III**	**Model IV**
AL	1.26(1.11,1.42)***	1.37(1.20,1.55)***	1.37(1.21,1.55)***	1.31(1.15,1.49)***
AK	1.23(1.03,1.48)*	1.28(1.06,1.54)**	1.28(1.06,1.54)**	1.21(1.00,1.46)*
AZ	1.19(0.98,1.43)	1.23(1.02,1.48)*	1.23(1.02,1.48)*	1.20(0.99,1.45)
AR	1.12(0.98,1.27)	1.18(1.04,1.35)*	1.18(1.03,1.35)*	1.12(0.98,1.29)
CA	1.12(1.02,1.23)*	1.19(1.08,1.31)***	1.20(1.09,1.32)***	1.15(1.05,1.27)**
CO	1.08(0.97,1.20)	1.11(0.99,1.24)	1.11(1.00,1.24)	1.11(0.99,1.25)
CT	1.16(1.05,1.29)**	1.23(1.11,1.36)***	1.23(1.11,1.36)***	1.18(1.06,1.32)**
DE	1.26(1.09,1.46)**	1.35(1.16,1.56)***	1.35(1.17,1.56)***	1.25(1.07,1.45)**
DC	1.30(1.12,1.50)***	1.38(1.20,1.60)***	1.39(1.20,1.61)***	1.32(1.14,1.53)***
FL	1.19(1.06,1.33)**	1.24(1.11,1.39)***	1.25(1.11,1.39)***	1.19(1.06,1.34)**
GA	1.16(1.02,1.32)*	1.24(1.08,1.42)**	1.24(1.09,1.42)**	1.17(1.02,1.35)*
HI	1.37(1.22,1.55)***	1.43(1.27,1.61)***	1.43(1.27,1.62)***	1.34(1.18,1.51)***
ID	1.14(1.02,1.27)*	1.20(1.08,1.34)**	1.21(1.09,1.35)***	1.18(1.05,1.32)**
IL	1.13(1.01,1.27)*	1.17(1.04,1.31)**	1.18(1.05,1.32)**	1.12(1.00,1.26)
IN	1.18(1.06,1.31)**	1.21(1.09,1.35)***	1.22(1.09,1.36)***	1.17(1.05,1.31)**
IA	1.10(0.98,1.24)	1.19(1.05,1.34)**	1.19(1.05,1.34)**	1.13(1.00,1.28)*
KS	1.11(1.01,1.22)*	1.14(1.04,1.26)**	1.15(1.04,1.26)**	1.10(1.00,1.22)
KY	1.12(1.02,1.24)*	1.24(1.12,1.37)***	1.23(1.12,1.37)***	1.22(1.10,1.35)***
LA	1.33(1.18,1.49)***	1.49(1.32,1.67)***	1.49(1.32,1.67)***	1.44(1.27,1.63)***
ME	1.12(1.01,1.25)*	1.19(1.06,1.32)**	1.19(1.06,1.33)**	1.11(0.99,1.25)
MD	1.24(1.11,1.39)***	1.29(1.16,1.45)***	1.30(1.17,1.45)***	1.24(1.11,1.39)***
MA	1.13(1.05,1.21)**	1.17(1.08,1.26)***	1.18(1.09,1.27)***	1.14(1.06,1.23)***
MI	1.19(1.08,1.32)***	1.24(1.12,1.37)***	1.25(1.12,1.38)***	1.19(1.07,1.32)**
MN	1.07(0.94,1.20)	1.09(0.96,1.23)	1.09(0.96,1.23)	1.07(0.94,1.21)
MS	1.16(1.03,1.32)*	1.24(1.09,1.40)**	1.23(1.08,1.40)**	1.16(1.02,1.33)*
MO	1.14(1.01,1.29)*	1.22(1.07,1.38)**	1.22(1.07,1.38)**	1.16(1.02,1.32)*
MT	1.10(0.97,1.24)	1.12(0.99,1.27)	1.13(1.00,1.27)	1.09(0.96,1.23)
NE	1.18(1.04,1.33)**	1.26(1.11,1.42)***	1.26(1.11,1.42)***	1.24(1.09,1.40)**
NV	1.08(0.91,1.28)	1.16(0.98,1.37)	1.16(0.98,1.38)	1.11(0.94,1.32)
NH	1.25(1.13,1.39)***	1.31(1.18,1.46)***	1.32(1.18,1.46)***	1.26(1.13,1.40)***
NJ	1.28(1.16,1.42)***	1.31(1.19,1.45)***	1.32(1.19,1.46)***	1.27(1.14,1.41)***
NM	1.23(1.10,1.36)***	1.26(1.13,1.40)***	1.26(1.13,1.41)***	1.22(1.09,1.36)***
NY	1.23(1.11,1.36)***	1.27(1.15,1.41)***	1.28(1.15,1.42)***	1.24(1.12,1.38)***
NC	1.16(1.04,1.29)**	1.28(1.15,1.43)***	1.29(1.15,1.43)***	1.19(1.06,1.33)**
ND	1.15(1.00,1.32)	1.21(1.05,1.39)**	1.21(1.06,1.39)**	1.17(1.01,1.35)*
OH	1.32(1.18,1.48)***	1.38(1.23,1.55)***	1.38(1.23,1.55)***	1.32(1.17,1.49)***
OK	1.42(1.29,1.56)***	1.45(1.32,1.60)***	1.43(1.30,1.58)***	1.36(1.23,1.51)***
OR	1.20(1.07,1.33)**	1.26(1.13,1.41)***	1.27(1.14,1.42)***	1.23(1.10,1.37)***
PA	1.29(1.16,1.42)***	1.34(1.21,1.48)***	1.34(1.22,1.48)***	1.30(1.18,1.44)***
RI	1.16(1.05,1.29)**	1.22(1.10,1.36)***	1.24(1.11,1.37)***	1.18(1.06,1.31)**
SC	1.29(1.14,1.46)***	1.38(1.23,1.56)***	1.39(1.23,1.56)***	1.35(1.19,1.53)***
SD	1.32(1.18,1.49)***	1.41(1.25,1.58)***	1.41(1.25,1.58)***	1.37(1.21,1.54)***
TN	1.18(1.04,1.34)**	1.20(1.06,1.36)**	1.19(1.05,1.36)**	1.16(1.01,1.32)*
TX	1.12(1.01,1.24)*	1.19(1.08,1.32)***	1.20(1.08,1.32)***	1.17(1.05,1.29)**
UT	1.12(0.99,1.26)	1.16(1.03,1.32)*	1.17(1.04,1.33)*	1.13(1.00,1.29)
VT	1.23(1.11,1.35)***	1.33(1.20,1.46)***	1.33(1.20,1.46)***	1.30(1.18,1.44)***
VA	1.24(1.07,1.43)**	1.31(1.14,1.52)***	1.32(1.14,1.53)***	1.27(1.10,1.48)**
WA	1.11(1.03,1.20)**	1.19(1.10,1.29)***	1.20(1.11,1.30)***	1.15(1.06,1.24)***
WV	1.04(0.93,1.16)	1.16(1.04,1.30)**	1.16(1.04,1.30)**	1.09(0.98,1.23)
WI	1.23(1.08,1.40)**	1.28(1.13,1.45)***	1.28(1.13,1.46)***	1.22(1.07,1.39)**
WY	1.13(1.01,1.27)*	1.16(1.04,1.30)*	1.17(1.04,1.31)**	1.11(0.99,1.25)
US	1.18(1.15,1.20)***	1.23(1.20,1.26)***	1.23(1.21,1.26)***	1.18(1.15,1.20)***

*p value less 0.05; **p value less 0.01; ***p value less 0.001.

## Discussion

Our findings indicate that current asthma prevalence among adults increased significantly from 2000–2001 to 2008–2009 for the majority of states (40/50) and for the U.S. as a whole. Our findings also suggest that place of residence, sociodemographic factors, and modifiable behaviors such as obesity might have contributed to those increases.

We considered population-based sociodemographic factors in the analysis because asthma prevalence is known to vary by certain population subgroups in the United States. Consistent with previous studies [8-12], asthma prevalence was higher among women than men and among non-Hispanic black than non-Hispanic white persons. Asthma was more prevalent among persons with less than a high school education than among persons with a bachelor degree, and also more prevalent among persons with an annual household income < $15,000 than among persons with a household income ≥ $75,000. Over the last century, the demographic changes for the nation in general reveal a decrease in the men-to-women ratio and an increase in minority populations [35]. These findings, coupled with the pattern of sociodemographic factors among adults with asthma, suggest that the increase in asthma prevalence among adults might be due, in part, to changes in population subgroups. The number of population subgroups with higher proportions of persons with asthma (e.g., women, non-Hispanic black persons) has been increasing at a higher rate than has subgroups with relatively lower proportions of persons with asthma (e.g., men, non-Hispanic white persons). When these changes were accounted for in the analysis, the number of states with a significant linear increasing trends in current asthma prevalence increased from 40 (80%) to 46 (92%) states, including 6 of the10 states (AR, AZ, IA, ND, UT, and WV) and D.C. that had insignificant increasing linear trends when state-specific changes in sociodemographic subgroups were not considered.

In this analysis, the number of states with significant increases in current asthma prevalence did not change when smoking status was added to the model with survey year and sociodemographic factors. It seems that the positive public health effects of tobacco control have become overshadowed by other factors contributing to the increase in current asthma prevalence in the last decade. However, when weight status was considered with survey year, sociodemographic factors, and smoking status, 10 states (AK, IL, KS, ME, WY, AR, AZ, IA, UT, and WV) that had previously shown a significant increase in current asthma prevalence when considering those variables showed no significant increase in prevalence. These findings suggest that the high prevalence of asthma among obese (10.2%, 95% CI 10.0-10.3) and morbidly obese (18.2%, 95% CI 17.7-18.7) persons, coupled with the increasing prevalence of obese persons in the United States [36], could contribute to observed increases in adult asthma prevalence in some states. An analysis comparing two cycles of data from the National Health and Nutrition Examination Survey (NHANES 2001–2002 and 2003–2004) similarly showed that adult asthma prevalence is increasing with greater prevalence among obese and morbidly obese adults [7].

Modifiable factors such as obesity and smoking have been associated with the development of asthma [13-20,22-25], decline in lung function, severity of asthma symptoms, and diminished response to steroid medication [37-42]. Although the pathophysiologic mechanisms remain unclear, genetic studies indicate that the relationship between asthma and obesity might be explained by independent and combined biologic pathways [42]. An international population-based cohort study that collected DNA samples from 9,167 participants found an independent association between obesity and asthma (OR 2.4, 95% CI 1.7-3.2) and a stronger, combined effect (OR 6.1, 95% CI 2.5-14.4) among nonatopic obese persons [42]. An increased risk of asthma onset and exacerbation has been found also among current or former smokers, especially among women and nonatopic adults [43]. In one study among nonatopic adults, 20% of adult-onset asthma was attributed to current smoking, suggesting that a large proportion of adult-onset asthma could be prevented by smoking cessation [43]. A population-based, nested, case–control study of adults (aged 21–51 years) in Sweden reported an increased risk for adult-onset asthma associated with noninfectious rhinitis that occurred before asthma onset (OR 5.4, 95% CI 4.0-7.2), especially among smoking nonatopic adults (OR 9.1, 95% CI 5.3-15.4), and among persons who smoked before asthma onset (OR 1.5, 95%CI 1.1-21) [44].

The factors accounted for in this analysis as well as other factors that could not be accounted for, such as changes in state public health policies, public and professional awareness of asthma symptoms, air pollution exposures, geographic locations (urban vs rural settings), and occupational exposures, might also be contributing to the observed increase in current asthma prevalence. Although the degree to which public and professional awareness has influenced asthma prevalence estimates could not be determined, the significant independent increase in current asthma prevalence by sociodemographic factors, smoking status, and weight status from this analysis suggests that the contribution might be relatively low, unless increased awareness and reporting of asthma symptoms follow similar patterns of variation. Substantial clinical and epidemiologic evidence suggest that indoor and outdoor air pollutants can increase asthma symptoms [45-53], and increasing evidence indicates that certain air pollutants can lead to asthma onset [54-59]. Although fewer studies on new onset asthma have been conducted among adults than children, several cohort studies have found that traffic-related local pollutants can contribute to the onset and manifestation of asthma in adults [55,58,59]. Also, a substantial proportion of adult-onset asthma has been attributed to occupational exposures [60-65]. In a population-based, 10-year prospective study of 6,837 adults (aged 20–44 years at study onset) from 13 countries that participated in the European Community Respiratory Health Surveys (ECRHS and ECRHS II) [66], Kogevinas et al. found large geographic variations in population attributable risk (PAR) for adult-onset asthma due to occupational exposures (PAR range 10-25%); significant excess risk for nurses (relative risk [RR] 2.2, 95% CI 1.3-4.0); and increased risk for participants who experienced an acute symptomatic inhalation event (e.g., fire, mixing cleaning products, or chemical spills) (RR 3.3, 95% CI 1.0-11.1) [61]. Another population-based cohort study of all employed adults aged 25–59 years in Finland from 1986 to 1998 found that a large proportion (400–500 new cases/million persons/year) of adult-onset asthma might have been attributable to occupational factors and, therefore, might have been prevented [64].

The results of this study are based on the analysis of a large representative population-based sample that was obtained by using standardized sampling methods and survey questions for all 50 states and D.C. over a 10-year period. However, three potential sampling frame errors that might have occurred due to nonresponse, noncoverage, and self-reported measures should be considered when interpreting the results. The median response rate for BRFSS each year was ~50%; however, asthma prevalence estimates in this study are similar to estimates in other studies with higher response rates, such as NHIS [67]. Noncoverage may occur because BRFSS does not survey institutionalized adults, the military, or residents without home telephones. We used BRFSS-weighted adjustments to minimize the effect of nonresponse and noncoverage. And, self-reported survey measurements may be less accurate than those based on physical measurements; however, a 1993 review of asthma questionnaires reported a sensitivity of 68% and specificity of 94% when self-reported asthma was compared to a clinical diagnosis of asthma [68].

## Conclusion

From 2000 to 2009, current asthma prevalence among adults increased for all 50 states and D.C., with significant increases in 46/50 (92%) states and D.C. After accounting for changes in population-based sociodemographic factors and smoking prevalence, the increasing prevalence of obese and morbidly obese persons in some states is associated with increasing prevalence of asthma, and if causal, could contribute to the increase.

### Next steps

Advances in asthma prevention and control are likely to originate from an understanding of population and environmental characteristics associated with temporal and geographic variations in asthma occurrence across states. Therefore, asthma surveillance systems that track these changes should be developed, maintained, and expanded within states and territories to support local-level analyses of population-based characteristics and environmental factors. State-by-state analysis is useful not only for public health planning but also for generating and testing hypotheses to explain the etiologic risk factors for asthma. Further study to identify changes in prevalence and their contributing risk factors at the state, county, and community level should be pursued to improve our understanding of asthma etiology, to identify high-risk groups, and to support state- and local-level intervention strategies to prevent asthma occurrence and control asthma symptoms.

## Competing interests

The authors declare that they have no competing interests.

## Authors’ contributions

All authors were responsible for the design and development of the study. XZ and TMC equally involved in all phases of the study including designing of the study, data collection and data analysis, and write-up of the manuscript. JBH and DBC participated in its research design and final drafting of the manuscript. All authors read and approved the final manuscript.

## Disclaimer

The findings and conclusions in this article are those of the authors and do not necessarily represent the official position of the Centers for Disease Control and Prevention.

## Pre-publication history

The pre-publication history for this paper can be accessed here:

http://www.biomedcentral.com/1471-2458/13/1156/prepub
